# Inhibition of Intestinal *α*-Glucosidase and Glucose Absorption by Feruloylated Arabinoxylan Mono- and Oligosaccharides from Corn Bran and Wheat Aleurone

**DOI:** 10.1155/2016/1932532

**Published:** 2016-03-17

**Authors:** Lovemore Nkhata Malunga, Peter Eck, Trust Beta

**Affiliations:** ^1^Department of Food Science, University of Manitoba, Winnipeg, MB, Canada R3T 2N2; ^2^Department of Human Nutrition, University of Manitoba, Winnipeg, MB, Canada R3T 2N2; ^3^Richardson Centre for Functional Foods & Nutraceuticals, University of Manitoba, Winnipeg, MB, Canada R3T 2N2

## Abstract

The effect of feruloylated arabinoxylan mono- and oligosaccharides (FAXmo) on mammalian *α*-glucosidase and glucose transporters was investigated using human Caco-2 cells, rat intestinal acetone powder, and* Xenopus laevis* oocytes. The isolated FAXmo from wheat aleurone and corn bran were identified to have degree of polymerization (DP) of 4 and 1, respectively, by HPLC-MS. Both FAXmo extracts were effective inhibitors of sucrase and maltase functions of the *α*-glucosidase. The IC50 for FAXmo extracts on Caco-2 cells and rat intestinal *α*-glucosidase was 1.03–1.65 mg/mL and 2.6–6.5 mg/mL, respectively. Similarly, glucose uptake in Caco-2 cells was inhibited up to 40%. The inhibitory effect of FAXmo was dependent on their ferulic acid (FA) content (*R* = 0.95). Sodium independent glucose transporter 2 (GLUT2) activity was completely inhibited by FAXmo in oocytes injected to express GLUT2. Our results suggest that ferulic acid and feruloylated arabinoxylan mono-/oligosaccharides have potential for use in diabetes management.

## 1. Introduction

The burden of diabetes mellitus is expected to increase by 55% in 2035. Globally, the cost of diabetes treatment was over US$ 612 billion in 2014. Diabetes is a chronic disease epitomised by high circulating plasma glucose. Type 1 diabetes is caused by deficiency in insulin excretion by pancreatic beta cells whereas type 2 diabetes is a result of organs insensitivity to insulin. The normal blood glucose level is about 4 mM but increases to about 12 mM within 30 minutes after intake of high carbohydrate diet [1, 2]. High glucose concentration triggers secretion of insulin hormone which makes the liver take up excess glucose for glycogen synthesis (glucose storage) and increase uptake of glucose by muscle cells through activation of GLUT4 [3]. Lapse in insulin response or production results in diabetes [4]. Thus management of postprandial glucose is critical in prevention and treatment of type 2 diabetes patients. Decrease in postprandial hyperglycemia can be attained by limiting intestinal carbohydrate digestion or uptake.

Starch and sucrose are the most common sources of dietary carbohydrates. Starch is digested primarily to maltose and other short chain carbohydrates by salivary and pancreatic amylase [5]. Human maltase-glucoamylase (MGAM) and sucrase-isomaltase (SI) [6] are the small intestinal brush border glucosidases responsible for final digestion of dietary carbohydrates prior to their absorption. Sugar absorption in the small intestine mainly involves GLUT2, GLUT5, and SGLT1 transporters [7–9]. The predominant glucose transporter in the intestine is GLUT2 as it is involved in both uptake and export [10]. SGLT1 and GLUT5 are expressed mostly on the apical side of enterocyte whereas GLUT2 is usually found on the basolateral membrane [11]. Glucose and galactose (at low concentration) are absorbed through SGLT1 whereas fructose is absorbed through GLUT5 [2]. The sugars are exported from the intestine through passive diffusion via GLUT2 and GLUT5 [2]. In case of high glucose and galactose levels in the lumen, SGLT1 facilitates insertion of GLUT2 on the apical membrane of the enterocyte to aid in glucose or galactose uptake [10]. Thus reduction of intestinal *α*-glucosidase activity and/or glucose absorption could effectively prevent or treat type 2 diabetes mellitus.

Consumption of whole grain cereals has been associated with a slow increase in blood glucose level compared to consumption of refined flours [12, 13] even though some results suggest otherwise [1]. The mechanism through which whole grains might reduce rapid increase in blood glucose is still not clear [14]. The presence of soluble dietary fiber is thought to negate starch hydrolysis and glucose absorption through increased viscosity. However, recent studies suggest that viscosity effect may be offset by strong intestinal peristalsis [15]. On the other hand, polyphenols have been reported to inhibit the activity of pancreatic amylase [16], intestinal *α*-glucosidase [17], and glucose transporters [18]. Phenolic compounds can bind to proteins, thereby affecting their functionality. Data on whether phenolic acids, which are abundant in cereals, can impair *α*-glucosidase and nutrient transporters is limited. Caffeic acid was reported to have mixed type inhibition potency towards *α*-amylase [16].

FA and its dimers are the most abundant phenolic acids in cereals and are mostly bound to arabinoxylan [19]. FA is concentrated in the outer layer of the cereal grains. Feruloylation happens at O-5 position of arabinose [20] substituted at O-2 and/or O-3 position of (1-4)-*β*-D-xylopyranose chain [21]. Feruloylated arabinose is the simplest feruloylated arabinoxylan present naturally in cereal and its quantity may increase by 2-fold following gastric digestion (unpublished data from our laboratory). Feruloyl arabinoxylan oligosaccharides (FOS) are also present in cereals but are mostly a product of enzymatic or acid hydrolysis of feruloylated arabinoxylan polysaccharides. FOS structures have been established and vary greatly in degree of polymerization and substitution [22–24]. FOS are a subject of interest because of their prebiotic and antioxidant potential [25]. At equal FA concentration, the FOS with higher DP tend to have higher antioxidant capacity [26]. Thus, we wanted to observe whether differences in DP would similarly affect their inhibition potencies towards intestinal *α*-glucosidase and glucose transporters. Hence our inclusion of FA, feruloylated arabinose, and FOS is carried out in our study.

We hypothesise that FA and FAXmo present in whole grain might contribute to decrease in postprandial plasma glucose by inhibiting activities of intestinal *α*-glucosidase and GLUT2 or SGLT1 transporter. Thus, we investigated the effect of FA, feruloylated arabinose (prepared from corn bran), and FOS (prepared from wheat aleurone) on mammalian intestinal *α*-glucosidase and intestinal glucose transporter.

## 2. Materials and Methods

### 2.1. Materials

Yellow corn was purchased locally from Bulk Barn (Winnipeg, Manitoba, Canada). A commercial wheat aleurone (GrainWise*™* wheat aleurone) was a gift from Cargill Limited's Horizon Milling (Wichita, Kansas, USA). [^3^H] 2-Deoxyglucose (25.5 Ci/mmol) was purchased from NEN Life Science Products (Boston, MA, USA). Dulbecco's modified Eagle medium (DMEM) (25 mM glucose) was obtained from Biofluids (Rockville, MD, USA) and all other media supplements were from Gibco Life Technologies (Gaithersburg, MD, USA). Caco-2 cells were purchased from American Type Culture Collection (Rockville, MD, USA). Acarbose, FA standard, sucrose, maltose monohydrate, D-glucose, D-xylose, D-arabinose, and D-galactose were purchased from Sigma-Aldrich (Milwaukee, Wisconsin, USA). All acids and organic solvents were obtained from Fischer Scientific (Whitby, Ontario, Canada). All chemicals used were of analytical grade.

### 2.2. Preparation of Feruloylated Arabinoxylan Mono- and Oligosaccharides Extracts

Feruloylated arabinose was prepared from corn bran using mild acid hydrolysis [27] and feruloylated arabinoxylan oligosaccharides were prepared from wheat aleurone using xylanase treatment [26]. Specifically, hand dissected corn bran was obtained according to a method by Ndolo and Beta [28]. Both wheat aleurone and corn bran flour were destarched and deproteinised according to a method by Malunga and Beta [26]. The treated corn bran flour (20 g) was suspended in 200 mL of 50 mM hydrochloric acid and heated at 100°C for 3 hours in a shaking water bath. The suspension was neutralised with 6 M ammonium hydroxide and centrifuged at 10000 g and 4°C for 10 minutes. The supernatant was freeze dried. Wheat aleurone flour was treated with endo-1,4-*β*-xylanase from* N. patriciarum* as described in Malunga and Beta [26].

The samples were reconstituted with 10 mL millique water and further purified on XAD2 column as described by Saulnier et al. [29]. XAD2 column was preconditioned with 1 column volume ethanol. After sample loading, it was eluted with 1 column volume water, 1.5 column volume methanol : water (50 : 50), and 1 column volume methanol. The 50% methanol eluent was collected and evaporated in rotary evaporator (40°C). The extracts were reconstituted with water and freeze dried.

### 2.3. Chemical Composition of Feruloylated Arabinoxylan Mono- and Oligosaccharides

Monosaccharide composition, FA content, and protein content were analyzed as described previously by Malunga and Beta [30]. Feruloylated arabinoxylan mono-/oligosaccharide species were identified by a reverse phased high performance liquid chromatography (Waters Alliance 2695 Instrument (Waters, Milford, MA)) system coupled to mass spectrometer (Q-TOF MS) (Micromass, Waters Corp., Milford, MA) as described by Malunga and Beta [26].

### 2.4. Cell Culture

Caco-2E stock cell cultures were maintained in 75 cm^2^ plastic flasks and cultured at 37°C in a 95% air, 5% CO_2_ atmosphere in Dulbecco's modified Eagle's minimal essential medium containing 15 mM glucose supplemented with 10% heat-inactivated FBS, 0.1 mM nonessential amino acids, and 0.1 mM glutamine. All experiments were carried out on cells of passage number 15 to 25 and media were refreshed 3 hours prior to experiments.

### 2.5. Inhibition of Mammalian *α*-Glucosidase Activity Assays

#### 2.5.1. Inhibition of *α*-Glucosidase Activity Assay in Caco-2 Cells

Confluent Caco-2 cell grown on 24-well plates were used for *α*-glucosidase inhibitory activity according to Pan et al. [31] with modifications. Briefly, 100% confluent cells were rinsed 3 times with PBS. Samples (350 *μ*L) dissolved in 10 mM HEPES buffer (pH 7.4, glucose free, 147.0 mM NaCl, 5.0 mM KCl, 1.9 mM KH_2_PO_4_, 1.1 mM Na_2_HPO_4_, 0.3 mM MgSO_4_-7H_2_O, 1.0 mM MgCl_2_-6H_2_O, and 1.5 mM CaCl_2_-2H_2_O) were added followed by 28 mM substrate (maltose or sucrose in HEPES buffer) (350 *μ*L). The cells were incubated at 37°C for 40 minutes. The substrate solutions were collected and boiled at 95°C for 10 minutes to deactivate enzyme activity. After centrifugation at 12000 rpm for 5 minutes, the supernatants were collected for glucose analysis using Megazyme glucose oxidase/peroxidase (GOPOD) glucose test kit. Cells were lysed with 10 mg CHAPS (3-((3-cholamidopropyl)dimethylammonio)-1-propanesulfonate) in 0.1 M NaOH (1 mL). The supernatant was analyzed for protein content using Lowry method. The data obtained was expressed as glucose per *μ*g protein (A). Alpha-glucosidase (sucrase or maltase) inhibition % was calculated as (1 − [(*A*_sample_ − *A*_blank_)/(*A*_control_ − *A*_blank_)]). IC50 (defined as the sample concentration resulting in 50% inhibition of *α*-glucosidase activity) was determined from the plot of %  *α*-glucosidase inhibition against sample concentration.

#### 2.5.2. Inhibition Assay for Rat Intestinal *α*-Glucosidase Activity

The *α*-glucosidase inhibitory method by Oki et al. (1999) [32] was used with modifications. Briefly, rat intestinal acetone powder (500 mg) was mixed with 10 mL sodium phosphate buffer (pH 6.9, 0.1 M) and sonicated in ice bath for 30 seconds (12 times) with 15-second break to prevent heat buildup. It was later centrifuged at 10000 g at 4°C for 10 minutes. The supernatant was collected and labeled rat intestinal *α*-glucosidase. Later, 50 *μ*L rat intestinal *α*-glucosidase was mixed with 100 *μ*L sample or buffer (control) and incubated at 37°C for 5 minutes. 50 *μ*L of 20 mM sucrose or 10 mM maltose was added and further incubated for 60 minutes (sucrose) or 30 minutes (maltose). The enzyme activity was stopped by heating to 95°C for 10 minutes. After centrifugation at 10000 g for 10 minutes, the supernatants were collected for glucose analysis using Megazyme GODP glucose test kit. Alpha-glucosidase (sucrase or maltase) inhibition % was calculated as (1 − [(*A*_sample_ − *A*_blank_)/(*A*_control_ − *A*_blank_)]). IC50 value was determined from the plot of %  *α*-glucosidase inhibition against sample concentration. Inhibition of rat intestinal *α*-glucosidase with acarbose (a known *α*-glucosidase inhibitor) was also done for comparison purpose. Acarbose concentrations of 1.625, 3.25, 4.9, 6.5, 9.8, and 13 *μ*g/mL were used instead of sample.

### 2.6. Assay for Glucose Uptake Inhibitory Activity in Caco-2 Cells

A method as described by Kwon et al. (2007) [18] was used with modifications. Confluent Caco-2 cells grown on 96-well plates were rinsed 3 times with PBS and incubated in preincubation buffer (HEPES buffer with 5 mM glucose) for 30 minutes at 37°C. After decanting, 50 *μ*L of HEPES buffer (pH 7.4, glucose free) containing sample was added followed by 50 *μ*L [^3^H] 2-deoxyglucose (5 mM in glucose-free HEPES buffer). The cells were incubated at room temperature for 15 minutes and transport activity was stopped by adding 100 *μ*L of ice cold preincubation buffer immediately after removal of transport buffer. Preincubation buffer was replaced with 60 *μ*L lysis buffer (10 mg CHAPS in 1 mL of 0.1 M NaOH) and incubated at room temperature for 2 hours. Aliquot was transferred to a scintillating vial for scintillation spectrometry (45 *μ*L) and protein (10 *μ*L) measurements. The glucose uptake was expressed as counts per minute beta (cpma) per *μ*g protein.

To study the effect of sample on glucose transporter 2 (GLUT2), glucose uptake studies were done similarly, but HEPES buffer without sodium was used instead.

### 2.7. Assay for Glucose Uptake in GLUT2 Injected Oocytes

Transport of glucose in oocytes from* Xenopus laevis was* done as described by Kwon et al. (2007). Briefly, oocytes were defolliculated by incubating the open ovarian lobes for 60 min at 23°C in OR-2 buffer without calcium (5 mM HEPES, 82.5 mM NaCl, 2.5 mM KCl, 1 mM MgCl_2_, 1 mM Na_2_HPO_4_, 100 *μ*g/mL gentamicin, and pH 7.8) containing 2 mg/mL collagenase (Sigma). Mature oocytes were isolated and kept at 18°C in OR-2 buffer containing 1 mM calcium chloride and 1 mM pyruvate. After 24 hours, the oocytes were injected with 36.8 nL cRNA (0.3 *μ*g/*μ*L) coding for GLUT2 or water using a Nanoject II injector (Drummond Scientific, Broomall, PA). The oocytes were incubated for 72 hours with media change every 12 hours.

FAXmo were dissolved in OR-2 containing calcium chloride without pyruvate at concentrations shown in the results section. Ferulic acid was first dissolved in dimethyl sulfoxide (DMSO) and diluted with OR-2 containing calcium chloride without pyruvate such that the final concentration of DMSO is less than 1%. Glucose transport was initiated by adding equal volumes (100 *μ*L) of treatment solutions and 2-[^3^H] deoxyglucose to 20 oocytes. OR-2 buffer was used as a control. After 30 minutes, transport buffer was aspirated and 1 mL ice cold OR-2 buffer was added to terminate glucose transport. The oocytes were washed four times with ice cold buffer. Each oocyte was put in antistatic pony scintillation vial (Perkin-Elmer, Canada) containing 200 *μ*L sodium dodecyl sulfate (10%). After 10 minutes, 5 mL scintillation cocktail was added prior to reading on scintillation spectrometry (Perkin-Elmer, Canada). The internalized glucose was expressed as counts per minute beta (cpma) per oocyte. Oocytes injected with water (instead of GLUT2 cRNA) were used to verify uptake of glucose in absence of GLUT2 cRNA.

### 2.8. Statistical Analysis

All cell culture experiments were done in three different generations of cells and data represents mean and standard deviation of at least six results that were similar. Data for rat intestinal *α*-glucosidase inhibition studies are mean and standard deviation of triplicate analyses. Each data point for glucose uptake in oocytes represents mean of 15–20 oocytes. All statistics were calculated using one-way analysis of variance (ANOVA) on JMP 10 statistical software (SAS Institute Inc., Cary, NC). Sample means were compared using Tukey's HSD method and significant differences determined at *p* ≤ 0.05. Correlations between FAXmo and inhibition capacity were done by Pearson's correlation test.

## 3. Results and Discussion

### 3.1. Isolation and Identification of Feruloylated Arabinoxylan Mono- and Oligosaccharides

**Figure 1:**
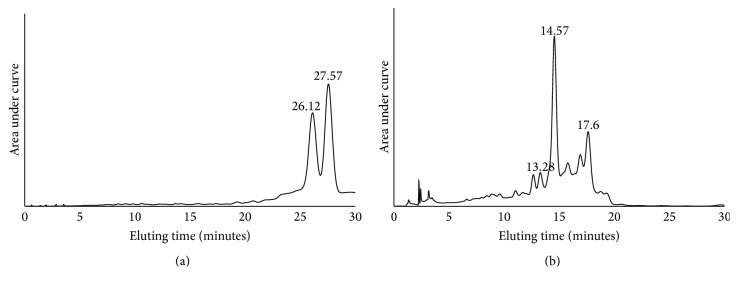
HPLC chromatograms of feruloyl mono- and oligosaccharide arabinoxylans from corn bran (a) and wheat aleurone (b).

**Figure 2:**
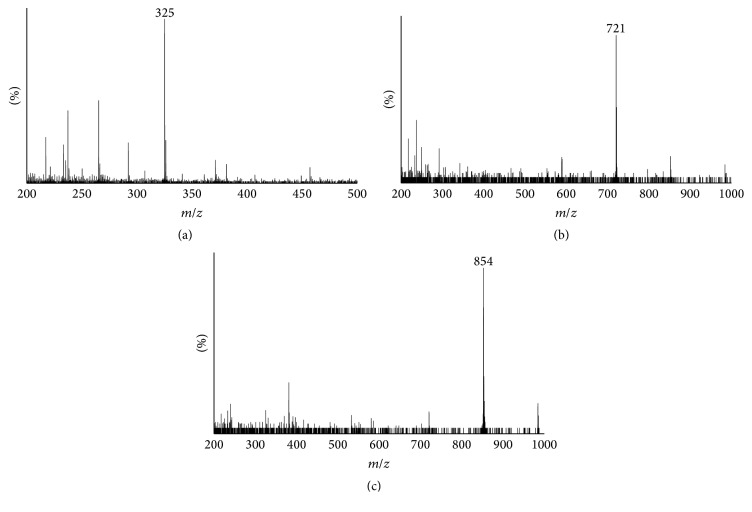
Negative ion mass spectra of feruloyl mono/oligosaccharide arabinoxylans [M-H]^−^ from corn bran (a) and wheat aleurone (b and c). Compounds eluting at 26.12 and 27.57 minutes in corn bran UV spectra had the same molecular weight [M-H]^−^ of 325 (a). Also compounds eluting at 13.28 minutes in wheat aleurone UV spectra had *m*/*z* = 721 (b) whereas those at 14.57, 15.78, 16.92, and 17.63 minutes had the same *m*/*z* = 853 (c).

**Figure 3:**
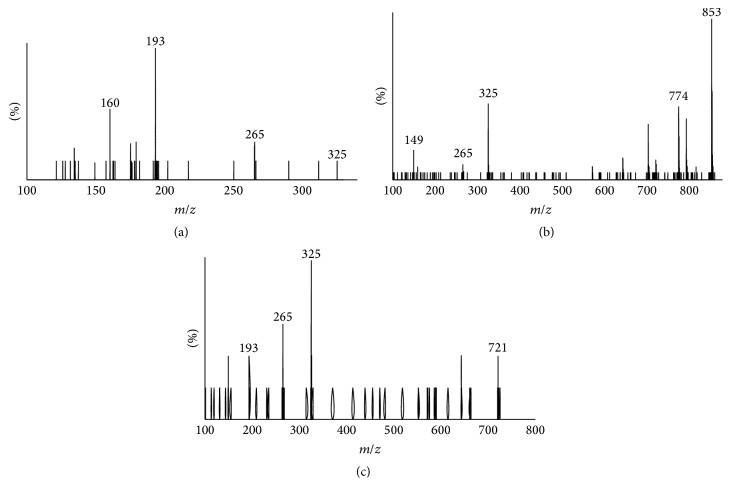
MSMS spectra of feruloyl mono- and oligosaccharide arabinoxylans [M-H]^−^ from corn bran and wheat aleurone: (a) *m*/*z* = 325; (b) *m*/*z* = 853; and (c) *m*/*z* = 721.

We aimed at obtaining feruloylated arabinoxylan extracts rich in feruloyl arabinose or FOS. FAXmo extracts from corn bran had about 19% protein, 9.85% FA, and 65.9% carbohydrate by weight on dry basis. The monosaccharide constituents in the carbohydrate fraction were arabinose (52.87%), xylose (42.95%), glucose (2.13%), mannose (1.07%), and galactose (0.97%). FAXmo extract from wheat aleurone was characterized to contain 29% protein, 7.2% FA, and 59.4% total carbohydrates. The molar percent distribution of the wheat bran FAXmo for arabinose, xylose, glucose, mannose, and galactose was 26.15, 65.15, 5.23, 2.12, and 1.36%, respectively. The HPLC chromatograms for corn bran (Figure 1(a)) and wheat aleurone (Figure 1(b)) FAXmo showed that the extracts contained different compounds. The negative ESI mass spectra for FAXmo in corn and wheat aleurone extracts showed a compound with *m*/*z* = 325 and *m*/*z* = 721, 854, and 985, respectively (Figure 2). The results suggest that corn bran FAXmo extract contained feruloyl arabinose (*M*_*w*_ = 326) and wheat aleurone extract was a heterogeneous FOS consisting of FA esterified to an arabinoxylan with 4 or 5 sugar moieties with the majority being 5. Fragmentation of the deprotonated ion *m*/*z* = 325 yielded daughter ions with *m*/*z* = 265, 193, and 134 (Figure 3(a)) typifying 5-*O*-feruloyl-L-arabinofuranosyl. Feruloyl arabinose was also produced through acid hydrolysis of corn bran [33]. Fragmentation of compound (*m*/*z* = 853) resulted in daughter ions 775, 721, 643, 325, and 265 (Figure 3(b)) which is similar to that reported by Wang et al. [34] extracted from wheat bran. They concluded that the structure was* O*-*β*-d-xylopyranosyl-(1→4)-*O*-[5-*O*-(feruloyl)-*α*-l-arabinofuranosyl-(1→3)]-*O*-*β*-d-xylo-pyranosyl-(1→4)-*O*-*β*-d-xylopyranosyl-(1→4)-d-xylopyranose following NMR analysis. Likewise the fragmentation pattern of compound with *m*/*z* = 721 (Figure 3(c)) matched that of* O*-*β*-d-xylopyranosyl-(1→4)-*O*-[5-*O*-(feruloyl)-*α*-l-arabinofuranosyl-(1→3)]-*O*-*β*-d-xylopyranosyl-(1→4)-d-xylo-pyranose [34].

### 3.2. Effect of Ferulic Acid and Feruloylated Arabinoxylan Mono- and Oligosaccharide Extracts on Mammalian *α*-Glucosidase

The data of the effect of FAXmo extract and FA on *α*-glucosidase are presented as IC50 value (Table 1). The results suggest that both FAXmo extracts and FA can inhibit *α*-glucosidase activity in a dose dependent manner. In Caco-2 cells, corn bran FAXmo showed a higher potency towards both sucrase and maltase activity inhibition compared to that of wheat aleurone FAXmo extracts. The inhibitory potency of FAXmo was highly correlated to FA content (*R* = 0.95). Similarly, at equal FA concentration (0.04 mg/mL), FA and FAXmo from corn bran and wheat aleurone inhibited sucrase activity by 18.70 ± 5.25, 23.28 ± 4.51%, and 19.45 ± 4.85%, respectively. Also, IC50 values based on FA were not significantly different (*p* < 0.05) for FA (0.09 mg/mL) and for FAXmo from corn bran (0.10 mg/mL) and wheat aleurone (0.09 mg/mL). Thus FA suppressed intestinal *α*-glucosidase activity in both free and bound forms. Acarbose (a known *α*-glucosidase inhibitor) was 75 times more potent compared to FA (Table 1(b)). Acarbose is currently used as drug for type 2 diabetic mellitus. However, FAXmo are consumed in large quantities as part of the dietary fiber.

Phenolic compounds have been reported to noncompetitively inhibit *α*-glucosidase activity [17, 35]. Phenolic compounds bind to the enzyme complex, thereby suppressing its activity [36]. Activity of the sucrase-isomaltase complex can be partially or completely suppressed depending on the nature of phenolic compound present. Anthocyanins only inhibited maltase activity of the sucrase-isomaltase complex and had no effect on sucrase [37]. The inhibition potency of some polyphenol extracts was much higher for sucrase than that of maltase [17]. In our study, the IC50 values for maltase and sucrase activity were not significantly different (*p* < 0.05) suggesting that both were equally suppressed. Both D-xylose [38] and L-arabinose [39] may inhibit sucrase activity. Thus we anticipated FAXmo extracts to have higher inhibitory effect compared to FA. Our results suggest that, at the same FA concentration, arabinose or xylose did not affect the inhibition potency of FA towards *α*-glucosidase activity in Caco-2 cells. In contrast, esterification to arabinose and/or xylose reduced the inhibition potency of FA by 50% in rat intestine *α*-glucosidase activity.

### 3.3. Effect of Ferulic Acid and Feruloylated Arabinoxylan Mono- and Oligosaccharide on Glucose Uptake in Caco-2 Cells

**Figure 4:**
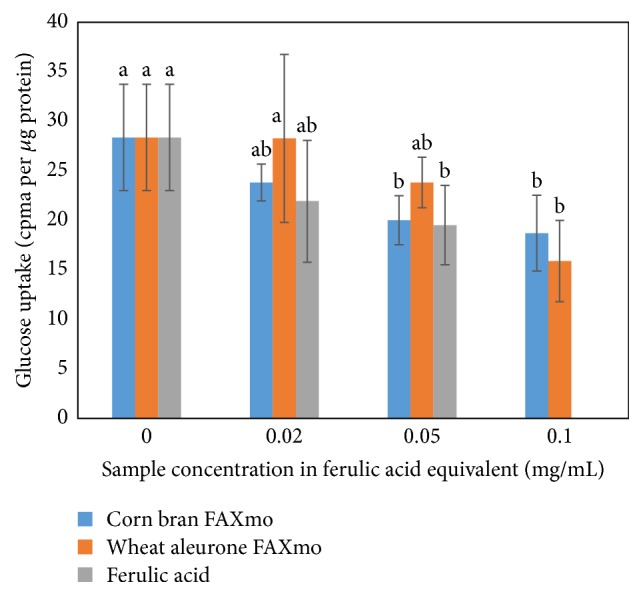
Effect of ferulic acid and feruloylated arabinoxylan mono- and oligosaccharides extracts on glucose uptake in Caco-2 cells in sodium plus medium. Values are presented as mean ± standard deviation (*n* = 6). Data with the same superscript are not significantly different at *p* ≤ 0.05. FAXmo means feruloylated arabinoxylan mono- and oligosaccharide extract.

We studied the effect of FA and FAXmo on glucose uptake in the presence of sodium. Our results (Figure 4) suggest that FA and FAXmo inhibit glucose uptake in Caco-2 cells. On weight basis, FA was the most potent inhibitor followed by feruloyl arabinose-rich extract suggesting that FA is the active site in FAXmo. Adjusting for FA content, the % inhibition potential of FA, feruloyl arabinose, and FOS on glucose uptake was not significantly different (*p* ≤ 0.05). Therefore, we concluded that FA can inhibit glucose uptake whether in free or bound form. Glucose is the predominant end product of dietary carbohydrate digestion. Glucose is absorbed in the small intestine via the sodium glucose linked transporter (SGLT1) and sodium independent glucose transporter 2 (GLUT2) expressed on the apical side of the intestinal epithelial cells.

**Figure 5:**
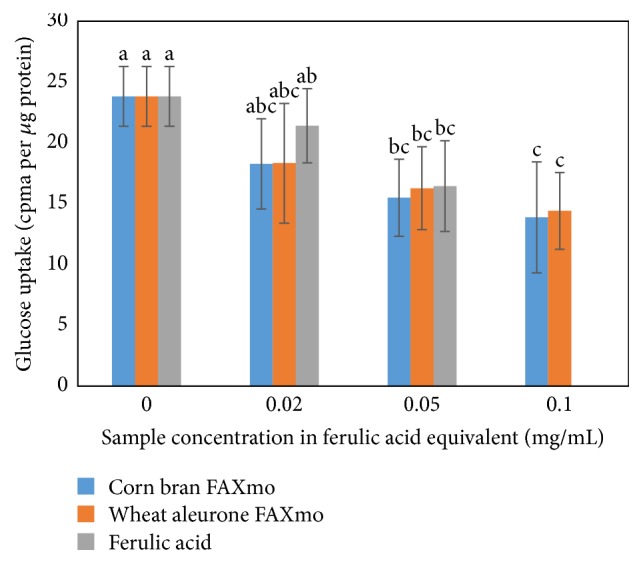
Effect of ferulic acid and feruloylated arabinoxylan mono- and oligosaccharides extracts on glucose uptake in Caco-2 cells in sodium-free medium. Values are presented as mean ± standard deviation (*n* = 6). Data with the same superscript are not significantly different at *p* ≤ 0.05. FAXmo means feruloylated arabinoxylan mono- and oligosaccharide extract.

We also conducted glucose uptake studies in the absence of sodium in order to understand which of the two transporters is being inhibited by FA and FAXmo. In the absence of sodium, glucose uptake was equally inhibited (Figure 5) suggesting that FA and FAXmo interfere with GLUT2 activities. GLUT2 is expressed on both the apical and basolateral sides of the intestinal epithelial cells. On the basolateral side, it functions to export glucose into the circulatory system. FA is well absorbed in the small intestine and consumption of arabinoxylan increases the concentration of plasma FA. Thus inhibition of GLUT2 by FA and FAXmo can be effective in attenuating postprandial hyperglycemia because it will decrease both intestinal glucose import and export. On the other hand, SGLT1 is arguably the chief glucose importer in the intestine when luminal concentrations are low. However, in case of high glucose and galactose levels in the lumen, SGLT1 facilitates insertion of GLUT2 on the apical membrane of the enterocyte to aid in glucose or galactose uptake [10]. It was interesting to note that absence of sodium did not significantly affect the amount of glucose uptake in absence of inhibitors. This observation is consistent with reports suggesting that SGLT1 does not transport 2-deoxyglucose [18]. This could explain why the amount of glucose internalized by cells in sodium-free buffer was not significantly different compared to when sodium was present during our Caco-2 studies.

**Figure 6:**
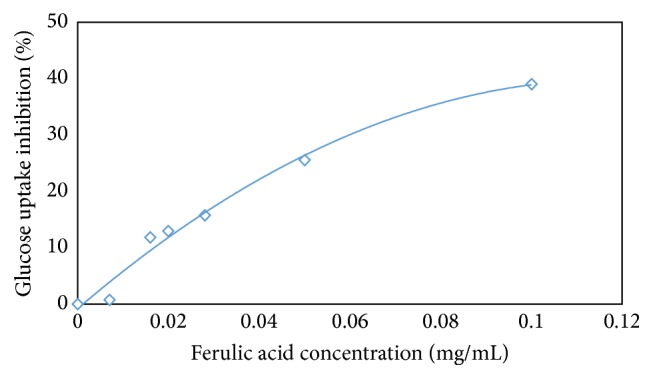
Glucose uptake inhibition (%) by ferulic acid in Caco-2 cells. A plot to demonstrate that ferulic acid is the active site for inhibiting glucose absorption in Caco-2 cells. Inhibition percent was calculated from means of glucose uptake in the presence of ferulic acid or ferulic acid sugar esters.

Several flavonoids have been reported to inhibit only GLUT2 but not SGLT1 [18]. Our results suggest that one of the most efficacious inhibitors, quercetin (78%), is 4 times more potent than FA at the same concentration. A plot of FAXmo's FA concentration against % glucose uptake inhibition (Figure 6) provides an insight of inhibition type. At very low concentrations, the % inhibition is proportional to increase in FAXmo concentration. However, as the concentration of FAXmo increases beyond 200 *μ*M FA equivalent the change in inhibition % becomes less noticeable. It appears that the maximum inhibition % attainable is 40% at our experimental conditions. This may suggest a noncompetitive inhibition behavior where FA binds to transporter protein on nonactive site. Phenolic acid or polyphenols in general have the ability to bind to proteins.

### 3.4. Effect of Ferulic Acid and Ferulic Acid Sugar Esters on Glucose Uptake in Oocytes

In order to verify inhibition potency of FAXmo or FA towards GLUT2, oocytes injected with human GLUT2 cRNA were used to study glucose transport. The glucose uptake by oocytes injected with GLUT2 was 127.92 ± 10.29 cpma/oocyte in the absence of inhibitors. However, glucose uptake was zero in the presence of FAXmo or FA at different concentrations (100, 150, and 200 *μ*M ferulic acid equivalent). Our results suggest that glucose uptake was completely blocked in the presence of FA or FAXmo (≥100 *μ*M). Thus confirming that FAXmo inhibits GLUT2 as observed in Caco-2 studies. We noted that the % maximum inhibition differed between oocytes (100%) and Caco-2 (~40%) glucose uptake studies. Glucose uptake studies in Caco-2 cells are usually nonconclusive due to the presence of other glucose transporters [40]. In particular, Caco-2 being cancer cells expresses GLUT1 and GLUT3 (in addition to SGLT1, GLUT2, and GLUT5) to maximize glucose uptake unlike normal intestinal cells [40]. Therefore, it is probable that GLUT2 contributed to about 40% of total glucose uptake in Caco-2 cells in the absence of SGLT1 activity. Thus, upon nullifying GLUT2 activity, increase in FAXmo concentration did not increase the percent inhibition of glucose uptake beyond 40% in Caco-2 cells. On the other hand, oocytes are method of choice in establishing specificity of glucose uptake inhibitors because defolliculated oocytes are devoid of glucose transporters. Thus injection of human GLUT2 cRNA into oocytes guarantees that any absorbed glucose is a consequent of the expressed GLUT2.

Thus we conclude that FA and feruloyl mono/oligosaccharides may contribute to the antihyperglycemic properties of whole grain cereals. Studies on postprandial glucose levels after consumption of whole grain diet have yielded inconsistent data. This could be in part due to intra/interspecies variation in cereal grains phenolic acid content [41–43]. On average, corn has 4 times more FA content compared to wheat [43] despite having approximately the same starch content. Also, FA content within wheat ranges from 162 to 721 *μ*g/g [44]. On the other hand, wheat endosperm has been reported to have about 120 *μ*g/g [41]. Hence, it is difficult to assess the role of phytochemicals in type 2 diabetes based on epidemiological studies where food frequency questionnaires are being used. Therefore, assuming 2 L gastric volume, we can extrapolate from our data that daily consumption of 77 mg of FA (free or bound to mono/oligosaccharide) may effectively suppress postprandial plasma glucose levels. Whether dietary uptake of 77 mg of FA can be achieved depends largely on the type of cereal grain and cultivar. For example, consumption of 100 g whole grain corn or wheat can provide about 80 mg or 23 mg FA (free or bound to mono/oligosaccharide), respectively (unpublished data from our lab).

On the other hand, low water solubility of FA, 0.6 mg/mL [45], may present another challenge towards bioaccessibility under physiological conditions. The solubility of FA can increase to 10 mg/mL when FA is esterified to arabinose [33]. Arabinoxylan oligosaccharides (AXOS) are getting attention for their antioxidant and prebiotic properties. Thus use of AXOS high in feruloyl arabinoxylan mono/oligosaccharides content might be beneficial in management or prevention of type II diabetes.

## 4. Conclusion

In this work, we have demonstrated for the first time that feruloylated arabinoxylan mono/oligosaccharide inhibit mammalian intestinal *α*-glucosidase and glucose transporters. Both sucrase and maltase activities were equally inhibited. GLUT2 was inhibited in the presence of feruloylated arabinoxylan mono/oligosaccharide. FA moiety of feruloyl arabinoxylan mono/oligosaccharide was the active site for the observed inhibitory activity. These results may partly explain the antihyperglycemic properties of whole grain as both FA and feruloyl arabinoxylan mono/oligosaccharide are present in significant quantities in cereals.

## Figures and Tables

**Table 1a:** (a) Caco-2 cells *α*-glucosidase

Extract	IC50 (mg/mL)	
	Sucrose	Maltose
Corn bran FAXmo	1.03 ± 0.04^b^ (0.10)	1.65 ± 0.27^a^ (0.16)
Wheat aleurone FAXmo	1.28 ± 0.05^a^ (0.09)	1.34 ± 0.05^b^ (0.09)
Ferulic acid	0.09 ± 0.01^c^ (0.09)	0.08 ± 0.00^c^ (0.08)

**Table 1b:** (b) Rat intestinal *α*-glucosidase

Extract	IC50 (mg/mL)	
	Sucrose	Maltose
Corn bran FAXmo	2.66 ± 0.64^b^ (0.27)	4.8 ± 0.36^b^ (0.48)
Wheat aleurone FAXmo	6.43 ± 0.83^a^ (0.45)	5.08 ± 0.01^a^ (0.35)
Ferulic acid	0.22 ± 0.13^c^ (0.22)	0.22 ± 0.00^c^ (0.22)
Acarbose	0.005 ± 0.00^d^	0.003 ± 0.00^d^

Values are presented as mean ± standard deviation (*n* = 3 for rat intestinal *α*-glucosidase and *n* = 6 for Caco-2 studies). Data in the same column with the same superscript are not significantly different at *p* ≤ 0.05. Data in parenthesis are IC50 values in ferulic acid equivalent (mg/mL). IC50 value is the sample concentration resulting in 50% inhibition of *α*-glucosidase activity. FAXmo means feruloylated arabinoxylan mono- and oligosaccharide extracts.

## References

[1] Jenkins D. J. A., Wolever T. M. S., Taylor R. H., Barker H. M., Fielden H., Gassull M. A. (1981). Lack of effect of refining on the glycemic response to cereals. *Diabetes Care*.

[2] Wright E. M., Martín M. G., Turk E. (2003). Intestinal absorption in health and disease—sugars. *Bailliere's Best Practice & Research in Clinical Gastroenterology*.

[3] Vaulont S., Vasseur-Cognet M., Kahn A. (2000). Glucose regulation of gene transcription. *The Journal of Biological Chemistry*.

[4] Reaven G. M., Bernstein R., Davis B., Olefsky J. M. (1976). Nonketotic diabetes mellitus: insulin deficiency or insulin resistance?. *The American Journal of Medicine*.

[5] Dona A. C., Pages G., Gilbert R. G., Kuchel P. W. (2010). Digestion of starch: *in vivo* and *in vitro* kinetic models used to characterise oligosaccharide or glucose release. *Carbohydrate Polymers*.

[6] Sim L., Willemsma C., Mohan S., Naim H. Y., Pinto B. M., Rose D. R. (2010). Structural basis for substrate selectivity in human maltase-glucoamylase and sucrase-isomaltase N-terminal domains. *The Journal of Biological Chemistry*.

[7] Burant C. F. (1992). Fructose transporter in human spermatozoa and small intestine is GLUT5. *Journal of Biological Chemistry*.

[8] Davidson N. O., Hausman A. M. L., Ifkovits C. A. (1992). Human intestinal glucose transporter expression and localization of GLUT5. *The American Journal of Physiology—Cell Physiology*.

[9] Wright E. M., Loo D. D. F., Panayotova-Heiermann M. (1994). ‘Active’ sugar transport in eukaryotes. *Journal of Experimental Biology*.

[10] Kellett G. L., Brot-Laroche E. (2005). Apical GLUT2: a major pathway of intestinal sugar absorption. *Diabetes*.

[11] Wright E. M., Hirayama B. A., Loo D. F. (2007). Active sugar transport in health and disease. *Journal of Internal Medicine*.

[12] Foster-Powell K., Holt S. H., Brand-Miller J. C. (2002). International table of glycemic index and glycemic load values: 2002. *The American Journal of Clinical Nutrition*.

[13] Ludwig D. S., Eckel R. H. (2002). The glycemic index at 20 y. *The American Journal of Clinical Nutrition*.

[14] Belobrajdic D. P., Bird A. R. (2013). The potential role of phytochemicals in wholegrain cereals for the prevention of type-2 diabetes. *Nutrition Journal*.

[15] Dhital S., Dolan G., Stokes J. R., Gidley M. J. (2014). Enzymatic hydrolysis of starch in the presence of cereal soluble fibre polysaccharides. *Food & Function*.

[16] Narita Y., Inouye K. (2009). Kinetic analysis and mechanism on the inhibition of chlorogenic acid and its components against porcine pancreas *α*-amylase isozymes I and II. *Journal of Agricultural and Food Chemistry*.

[17] Zhang H., Wang G., Beta T., Dong J. (2015). Inhibitory properties of aqueous ethanol extracts of propolis on alpha-glucosidase. *Evidence-Based Complementary and Alternative Medicine*.

[18] Kwon O., Eck P., Chen S. (2007). Inhibition of the intestinal glucose transporter GLUT2 by flavonoids. *The FASEB Journal*.

[19] Ishii T. (1997). Structure and functions of feruloylated polysaccharides. *Plant Science*.

[20] Bunzel M., Ralph J., Steinhart H. (2005). Association of non-starch polysaccharides and ferulic acid in grain amaranth (*Amaranthus caudatus* L.) dietary fiber. *Molecular Nutrition and Food Research*.

[21] Lequart C., Nuzillard J.-M., Kurek B., Debeire P. (1999). Hydrolysis of wheat bran and straw by an endoxylanase: production and structural characterization of cinnamoyl-oligosaccharides. *Carbohydrate Research*.

[22] Gruppen H., Hoffmaann R. A., Kormelink F. J. M., Voragen A. G. J., Kamerlin J. P., Vliegenthart J. F. G. (1992). Characterisation by 1H NMR spectroscopy of enzymically derived oligosaccharides from alkali-extractable wheat-flour arabinoxylan. *Carbohydrate Research*.

[23] Katapodis P., Vardakou M., Kalogeris E., Kekos D., Macris B. J., Christakopoulos P. (2003). Enzymic production of a feruloylated oligosaccharide with antioxidant activity from wheat flour arabinoxylan. *European Journal of Nutrition*.

[24] Yuan X., Wang J., Yao H. (2006). Production of feruloyl oligosaccharides from wheat bran insoluble dietary fibre by xylanases from *Bacillus subtilis*. *Food Chemistry*.

[25] Ou J., Sun Z. (2014). Feruloylated oligosaccharides: structure, metabolism and function. *Journal of Functional Foods*.

[26] Malunga L. N., Beta T. (2015). Antioxidant capacity of arabinoxylan oligosaccharide fractions prepared from wheat aleurone using *Trichoderma viride* or *Neocallimastix patriciarum* xylanase. *Food Chemistry*.

[27] Allerdings E., Ralph J., Steinhart H., Bunzel M. (2006). Isolation and structural identification of complex feruloylated heteroxylan side-chains from maize bran. *Phytochemistry*.

[28] Ndolo V. U., Beta T. (2013). Distribution of carotenoids in endosperm, germ, and aleurone fractions of cereal grain kernels. *Food Chemistry*.

[29] Saulnier L., Vigouroux J., Thibault J.-F. (1995). Isolation and partial characterization of feruloylated oligosaccharides from maize bran. *Carbohydrate Research*.

[30] Malunga L. N., Beta T. (2015). Antioxidant capacity of water-extractable arabinoxylan from commercial barley, wheat, and wheat fractions. *Cereal Chemistry*.

[31] Pan G.-Y., Huang Z.-J., Wang G.-J. (2003). The antihyperglycaemic activity of berberine arises from a decrease of glucose absorption. *Planta Medica*.

[32] Oki T., Matsui T., Osajima Y. (1999). Inhibitory effect of *α*-glucosidase inhibitors varies according to its origin. *Journal of Agricultural and Food Chemistry*.

[33] Fang H.-Y., Wang H.-M., Chang K.-F. (2013). Feruloyl-l-arabinose attenuates migration, invasion and production of reactive oxygen species in H1299 lung cancer cells. *Food and Chemical Toxicology*.

[34] Wang J., Yuan X., Sun B., Cao Y., Tian Y., Wang C. (2009). On-line separation and structural characterisation of feruloylated oligosaccharides from wheat bran using HPLC-ESI-MSn. *Food Chemistry*.

[35] Shobana S., Sreerama Y. N., Malleshi N. G. (2009). Composition and enzyme inhibitory properties of finger millet (*Eleusine coracana* L.) seed coat phenolics: mode of inhibition of *α*-glucosidase and pancreatic amylase. *Food Chemistry*.

[36] Li Y., Gao F., Gao F., Shan F., Bian J., Zhao C. (2009). Study on the interaction between 3 flavonoid compounds and *α*-amylase by fluorescence spectroscopy and enzymatic kinetics. *Journal of Food Science*.

[37] Matsui T., Ueda T., Oki T., Sugita K., Terahara N., Matsumoto K. (2001). *α*-glucosidase inhibitory action of natural acylated anthocyanins. 1. Survey of natural pigments with potent inhibitory activity. *Journal of Agricultural and Food Chemistry*.

[38] Matsuura T., Ichikawa T. (1997). Evaluation of the sucrase inhibitory activity of D-xylose in rats with the catheterization of portal vein. *Journal of Japanese Society of Nutrition and Food Science*.

[39] Seri K., Sanai K., Matsuo N., Kawakubo K., Xue C., Inoue S. (1996). L-arabinose selectively inhibits intestinal sucrase in an uncompetitive manner and suppresses glycemic response after sucrose ingestion in animals. *Metabolism: Clinical and Experimental*.

[40] Mahraoui L., Rodolosse A., Barbat A. (1994). Presence and differential expression of SGLT1, GLUT1, GLUT2, GLUT3 and GLUT5 hexose-transporter mRNAs in Caco-2 cell clones in relation to cell growth and glucose consumption. *Biochemical Journal*.

[41] Ndolo V. U., Beta T. (2014). Comparative studies on composition and distribution of phenolic acids in cereal grain botanical fractions. *Cereal Chemistry*.

[42] Nyström L., Lampi A.-M., Andersson A. A. M. (2008). Phytochemicals and dietary fiber components in rye varieties in the HEALTHGRAIN diversity screen. *Journal of Agricultural and Food Chemistry*.

[43] Sosulski F., Krygier K., Hogge L. (1982). Free, esterified, and insoluble-bound phenolic acids. 3. Composition of phenolic acids in cereal and potato flours. *Journal of Agricultural and Food Chemistry*.

[44] Li L., Shewry P. R., Ward J. L. (2008). Phenolic acids in wheat varieties in the HEALTHGRAIN diversity screen. *Journal of Agricultural and Food Chemistry*.

[45] Mota F. L., Queimada A. J., Pinho S. P., Macedo E. A. (2008). Aqueous solubility of some natural phenolic compounds. *Industrial & Engineering Chemistry Research*.

